# Severe hepatopathy and neurological deterioration after start of valproate treatment in a 6-year-old child with mitochondrial tryptophanyl-tRNA synthetase deficiency

**DOI:** 10.1186/s13023-018-0822-6

**Published:** 2018-05-21

**Authors:** Elise Vantroys, Joél Smet, Arnaud V. Vanlander, Sarah Vergult, Ruth De Bruyne, Frank Roels, Hedwig Stepman, Herbert Roeyers, Björn Menten, Rudy Van Coster

**Affiliations:** 1 Department of Pediatric Neurology and Metabolism, Ghent University Hospital, Ghent, Belgium; 2 Center for Medical Genetics Ghent, Ghent University, Ghent, Belgium; 3 Department of Pediatric Gastroenterology, Hepatology and Nutrition, Ghent University Hospital, Ghent, Belgium; 4 Department of Pathology, Ghent University Hospital, Ghent, Belgium; 5 Department of Laboratory Medicine, Ghent University Hospital, Ghent, Belgium; 6 Department of Experimental Clinical and Health Psychology, Ghent University, Ghent, Belgium

**Keywords:** Mitochondrial tryptophanyl-tRNA synthetase, WARS2, Mitochondria, Mitochondrial aminoacyl-tRNA synthetase, Valproate, Hepatotoxicity, Cytochrome *c* oxidase, Mosaic

## Abstract

**Background:**

The first subjects with deficiency of mitochondrial tryptophanyl-tRNA synthetase (WARS2) were reported in 2017. Their clinical characteristics can be subdivided into three phenotypes (neonatal phenotype, severe infantile onset phenotype, Parkinson-like phenotype).

**Results:**

Here, we report on a subject who presented with early developmental delay, motor weakness and intellectual disability and who was considered during several years as having a non-progressive encephalopathy. At the age of six years, she had an epileptic seizure which was treated with sodium valproate. In the months after treatment was started, she developed acute liver failure and severe progressive encephalopathy. Although valproate was discontinued, she died six months later. Spectrophotometric analysis of the oxidative phosphorylation complexes in liver revealed a deficient activity of complex III and low normal activities of the complexes I and IV. Activity staining in the BN-PAGE gel confirmed the low activities of complex I, III and IV and, in addition, showed the presence of a subcomplex of complex V. Histochemically, a mosaic pattern was seen in hepatocytes after cytochrome *c* oxidase staining. Using Whole Exome Sequencing two known pathogenic variants were detected in *WARS2* (c.797delC, p.Pro266ArgfsTer10/ c.938 A > T, p.Lys313Met).

**Conclusion:**

This is the first report of severe hepatopathy in a subject with WARS2 deficiency. The hepatopathy occurred soon after start of sodium valproate treatment. In the literature, valproate-induced hepatotoxicity was reported in the subjects with pathogenic mutations in *POLG* and *TWNK*. This case report illustrates that the course of the disease in the subjects with a mitochondrial defect can be non-progressive during several years. The subject reported here was first diagnosed as having cerebral palsy. Only after a mitochondriotoxic medication was started, the disease became progressive, and the diagnosis of a mitochondrial defect was made.

## Background

The oxidative phosphorylation (OXPHOS) system is embedded in the inner mitochondrial membrane and consists of five complexes. Thirteen subunits of the OXPHOS complexes are encoded by mitochondrial DNA (mtDNA). A series of nuclear-encoded proteins are needed to carry out transcription and translation in the mitochondrial matrix. The latter are synthesized in the cytosol and have to be imported into the mitochondrial matrix. An important group among these are the mitochondrial aminoacyl-tRNA synthetases (mt-aaRSs) [1]. This is a well-described group of enzymes responsible for charging the mitochondrial-encoded tRNAs with their cognate amino acid. Defects in mt-aaRSs result in defective intramitochondrial translation, affecting mainly the OXPHOS complexes with the largest number of mitochondrial-encoded subunits, i.e. complex I and complex IV. The activity of complex II is normal, or even upregulated, as it is exclusively composed of nuclear-encoded subunits. The mitochondrial aaRSs differ from their cytoplasmic counterparts except for the enzymes encoded by *GARS* and *KARS* that take care of translation in the cytoplasm and in the mitochondria [1, 2].

DARS2 was the first mt-aaRS to be associated with a human disease [3]. During the last decade, molecular alterations in all nineteen mt-aaRSs have been linked to a heterogeneous group of human disorders affecting different organ systems. Mutations in a single gene can cause either a mild or severe phenotype, or even result in completely different phenotypes, as shown for *AARS2*, *NARS2* and *FARS2* [4–6]. The first observation of WARS2 deficiency has been reported by Musante et al. in 2017 [7]. *WARS2* codes for tryptophanyl-tRNA synthetase active in the mitochondria [8]. Three more papers describing WARS2 deficient subjects were published subsequently [9–11].

Here, we present a 6-year-old girl in whom severe delay of early cognitive and motor development and mild dysmorphic facial features were initially suggestive of cerebral palsy or a chromosomal disorder as she was slightly dysmorphic. Karyotyping and CGH-microarray were normal. It was only after sodium valproate was initiated that acute liver failure was diagnosed and the diagnosis of a mitochondrial disorder was suspected and confirmed.

## Methods

### Spectrophotometric analysis

Activity of citrate synthase and respiratory chain complexes I, II, II + III, III and IV were measured in liver homogenate using spectrophotometric analysis according to previously described methods [12].

### Blue native-polyacrylamide gel electrophoresis

Blue native polyacrylamide gel-electrophoresis (BN-PAGE) was used to separate and assay the activity of the five OXPHOS complexes. Mitochondria isolated from the proband’s liver and from controls were loaded in duplicate using equal amounts of mitochondrial protein (50 μg). Isolation and solubilization of the complexes, separation by BN-PAGE and staining of catalytic activities in the gel were performed as described earlier [13].

### Light microscopy, cytochemical analysis and electron microscopy of liver

Liver tissue was obtained by laparoscopy. One part was frozen for biochemical analysis and another part formalin-fixed and paraffin embedded. Hematoxylin and eosin staining and periodic acid-Schiff (PAS) staining before and after diastase digestion of glycogen were performed, as were stains of reticulin, immunostains using antibodies for cytokeratin-7 and Ki67, iron staining and staining with Sirius red.

For cytochrome *c* oxidase cytochemistry, liver tissue was fixed in cold glutaraldehyde 1% for two hours. After rinsing in saccharose 13%, frozen sections were stained for cytochrome *c* oxidase activity with diaminobenzidine at pH 6, as previously described [14]. Six µm sections were mounted after nuclear staining with light green. For electron microscopy, 60 μm sections were postfixed in OsO_4_ and embedded in epoxy resin. Semithin sections were studied by light microscopy. Ultrathin sections were counterstained with lead and studied in a Zeiss electron microscope.

### Whole exome sequencing

Exome enrichment and sequencing were performed by Aros AB (Aarhus, Denmark) using the Illumina TruSeq Exome Enrichment Kit (Illumina, San Diego, CA), followed by paired-end sequencing on a HiSeq 2000 (2 × 100 cycles). Data analysis including alignment to the GRCh37 human reference genome (NCBI), variant calling and variant filtering was done using the in-house developed analysis pipeline Seqplorer.

### Western blot analysis

Western blotting was performed using commercial antibodies against WARS2 (Sigma AV52366), VDAC-1 (Abcam, AB14734) and a cocktail of commercial antibodies directed against one subunit in each of the five OXPHOS complexes (NDUFB8 for complex I, SDHB for complex II, UQCRC2 for complex III, COX2 for complex IV and subunit alpha for complex V) (MS601 MitoProfile® Total OXPHOS Human WB Antibody Cocktail). Detection was performed using the ECL Plus™ enhanced chemiluminescence kit (GE Healthcare, Diegem, Belgium), as described previously [15]. A chemidoc-It © 500 Image System, UVP (Cambridge, UK) with a cooled charge-coupled device camera was used to capture the WARS2 signals. Image processing was performed using VisionWorksLQ image acquisition software. Biostep Celvin® S420 chemiluminescence imager from Biostep Gmbh ® (Burkhardtsdorf, Germany) equipped with a cooled charge-coupled device with a resolution of 4.2 Mpixel was used to capture the other signals. Image acquisition was achieved using the Celvin® SnapAndGo software and TotalLab for image analysis.

Whole cell lysates and mitochondrial fractions were prepared from skeletal muscle and heart muscle from controls. The same fractions were prepared from liver from the proband and controls. Electrophoretic separation was performed using tricine SDS-PAGE.

## Results

### Case report

The proband was born at term from non-consanguineous parents. Antenatal ultrasound examination revealed intra-uterine growth retardation. Birth weight was 2314 g, length 45 cm and head circumference 31.8 cm. The neonatal period was without complications. When examined at the age of eleven months, severe axial hypotonia, hypertonia and dystonic posturing of arms and legs were noticed and she was found to have slight dysmorphic features, i.e. a thin upper lip, low set ears, a broad nasal bridge, hypertelorism of the eyes and an ogival palatum. Eye movements were complete. Pyramidal tract signs were not found. Length was 67 cm (− 2.5 SD), weight 7.6 kg (− 2 SD) and head circumference 45 cm (0 SD). Brain MRI showed a relative small volume of both frontotemporal lobes, enlarged sulci and small zones with increased signal bilaterally on T2 weighted images in the frontal subcortical zones. When seen at eighteen months of age, a severe delay of cognitive and motor development was confirmed. The cognitive development was estimated at less than six months. She had no head control and rigidity of arms and legs was noticed. Fists were bilaterally clenched. Deep tendon reflexes were weak and both feet were in equinus position. At six years of age, she was hospitalized because of a long lasting episode with decreased awareness, lateral eye deviation and twitching of the eyelids at the left. Hypoglycemia and a slightly increased lactate (which turned out to be normal on the next day) were detected in serum on admission. Sodium valproate was started. One month later, she was taken to a local hospital after she was found stuporous at home in the morning. Dystonic posturing of the limbs together with motor hyperactivity and continuous choreatic movements were seen. Deep tendon reflexes were present although weak. Plantar reflexes were indifferent. Blood sugar concentration was 8 mg/dl (nl 60–130). In the course of two days, SGOT rapidly increased from 188 IU/L to 1000 IU/L (nl < 42) and SGPT from 159 to 487 IU/L (nl 5–25). Gamma-glutamyltransferase was 232 IU/L (nl 4–22). Concentration of sodium valproate in serum was 18 μg/ml (nl 50–90). On the second day of hospitalization, lactate in serum was high (5.5–12.8 mmol, nl < 1.8) and glycaemia was normalized. Urinary organic acid profile showed high concentrations of lactate (2686 μmol/mmol creatinine, nl < 131), pyruvate (66 μmol/mmol creatinine, nl < 17), 3-OH-butyrate (3161 μmol/mmol creatinine, nl < 7) and 3-keto-butyrate (2968 μmol/mmol creatinine, nl < 5). Acylcarnitine profile was normal. In CSF, cell count was not increased, glucose concentration was 66 mg/dl, protein 10 mg/dl and lactate 2.8 mmol (nl < 1.7). Coagulation parameters were perturbed (PT 34%, nl 70–120; aptt 51 s, nl 29–38), direct bilirubin was only slightly increased (0.41 mg/dL, nl 0–0.3) and indirect bilirubin was normal. Ammonemia was normal (34 μmol, nl 11–48). Sodium valproate was stopped immediately and phenobarbital was started. Ophthalmological examination revealed optic atrophy. EEG and electroneuromyographic examination were normal. Cerebral MRI showed enlargement of the lateral ventricles and third ventricle presumably due to white matter loss. Subarachnoidal spaces were abnormally large, most marked in the frontal regions bilaterally. In the posterior fossa, the vermis cerebelli was hypoplastic, the fourth ventricle was enlarged and the brainstem and cerebellar peduncles were hypoplastic. No signs of periventricular leucomalacia, neither of cystic encephalopathy were seen. She was discharged from the hospital. One month later, she was found stuporous and was referred now to the University Hospital. On arrival, she was icteric and the liver was borderline enlarged (1 cm below the costal margin). Transaminases were severely increased in serum (> 1000 IU/L). Coagulation parameters were perturbed. Direct bilirubin was 9 mg/dL and serum albumin was decreased (2.8 g/dL). Ammonemia was normal (36 μmol/L, nl 11–48). Lactate was 66 mg/dL (nl 9–16). She was diagnosed with acute liver failure (ALF) and metabolic encephalopathy. The urinary organic acid profile was suggestive for a defect in the oxidative phosphorylation. Plasma amino acid profile showed a slight increase of alanine, tyrosine and phenylalanine. In CSF, the lactate concentration was 2.8 mmol (nl < 1.8) and glucose 66 mg/dL (concomitant serum glucose 82 mg/dL). Echocardiogram was normal. Ultrasound examination of the liver showed increased reflectivity. She was discharged from hospital and a treatment was prescribed consisting of levetiracetam (380 mg/day), riboflavin (150 mg/day), thiamin (150 mg/day), pyridoxin (200 mg/day), co-enzyme Q (300 mg/day) and L-carnitine (1200 mg/day). Liver biopsy was performed via laparoscopy in ambulatory setting. During the next months, episodes with decreased awareness occurred more frequently. When seen ambulatory at the child neurology department, she was stuporous with ptosis of the eyelids bilaterally and severe generalized amyotrophy. No hepatomegaly neither signs of pyramidal tract involvement were found. Her neurological condition further deteriorated. Transaminases were measured repeatedly in blood and were either normal or only slightly increased. Ammonia in blood was also measured on several occasions and was normal. She passed away at home at the age of 6 ½ year.

### Light microscopy, cytochemical analysis and electron microscopy

**Fig. 1:**
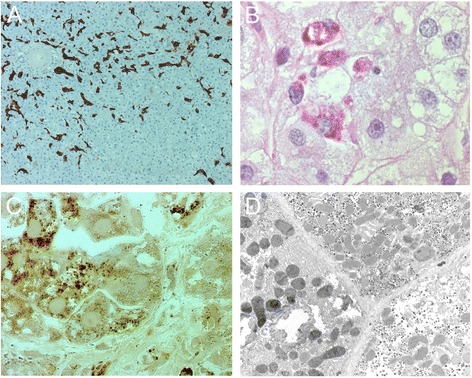
Light and electron microscopy. Legend: (**a)** Cytokeratin 7 immunostaining is positive in numerous small cells throughout the liver parenchyma. Some form abortive bile ducts. Their proliferation is proof of active liver regeneration. **b** Inclusions in macrophages indicate phagocytosis of debris from damage. PAS after diastase digestion of glycogen; nuclear counterstain with hematoxylin. Parenchymal cells at the right show large empty circular spaces, these are drops of dissolved fat. **c** Hepatocytes show mitochondria (rounded granules) with different degrees of brown reaction product of cytochrome *c* oxidase activity. Nuclei are unstained. 2 μm plastic section, after postosmication. Obj. 100×, oil immersion. **d** Mitochondrial mosaic observed by electron microscopy after reaction for cytochrome *c* oxidase activity. Mitochondria in the hepatocyte at left show dark reaction product in their cristae. Three unstained round profiles in this cell are peroxisomes. Also seen are two large fat globules that are partly dissolved. The other two hepatocytes have many mitochondria. Their cristae show little or no reaction product. The dark granules in the cytoplasm of all three cells are glycogen rosettes × 7000

The presence of Ki67 positive nuclei are proof of parenchymal cells proliferation, indicating regeneration after cell death. Macrophages (Kupffer cells) contained large PAS-diastase resistant inclusions, a consequence of parenchymal cell death (Fig. 1a). CK-7 positive small cells were numerous throughout the parenchyma sometimes forming abortive bile ducts (Fig. 1b). The latter are progenitor cells proliferating after parenchymal cell death. Lipid droplets were seen in some parenchymal cells.

Cytochrome *c* oxidase activity staining visualized a mosaic pattern. Hepatocytes with strong mitochondrial activity were adjacent to cells scarcely showing reaction product (Fig. 1c-d).

### Biochemical and molecular studies

**Fig. 2:**
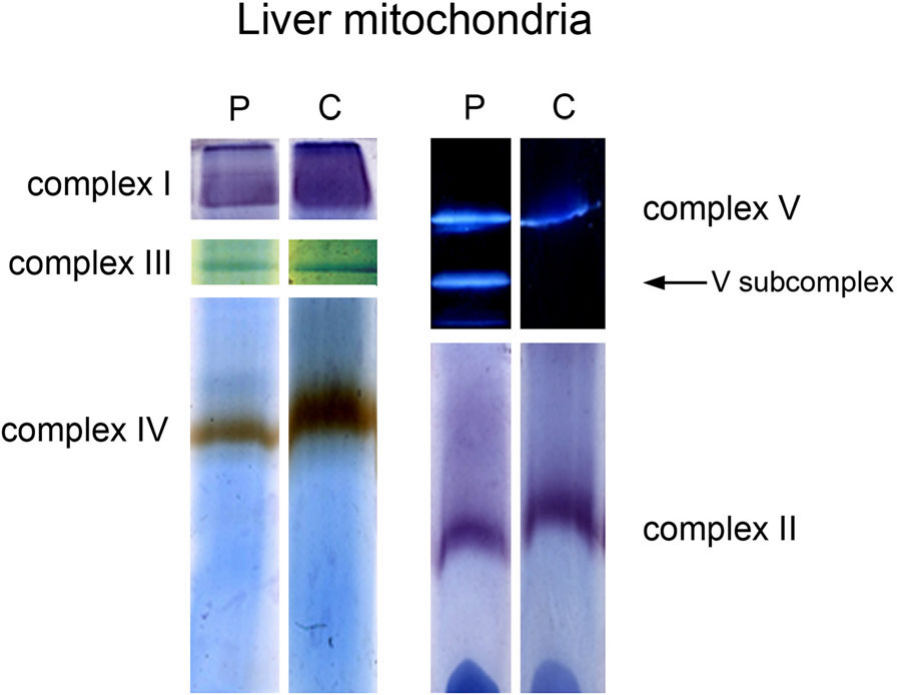
BN-PAGE followed by in-gel activity staining. Legend: In-gel activity staining of complex I, II, III, IV and V in liver tissue from the proband compared to a control revealed lower activity of complex I, III and IV in the proband. A catalytically active subcomplex of complex V was seen in the proband

Spectrophotometric analyses in liver tissue from the proband showed significantly reduced activity of complex III and low activities of complex I and IV (although still within normal control range) (Table 1). BN-PAGE confirmed the low activities of the complexes I, III and IV when compared to those in liver tissue from a control and, in addition, showed the presence of a catalytically active subcomplex of complex V (Fig. 2). These results were suggestive of a defect in replication, transcription or translation in the mitochondrial matrix. Mutations in mtDNA were excluded using whole mtDNA sequencing.

**Table 1:** OXPHOS activities in liver homogenate from the proband measured by spectrophotometric analysis

OXPHOS COMPLEXES	Activity (Control range)	Ratio/CS	Z- score
Complex I	12 (9–49)	0.50	−1.65
Complex II	135 (52–202)	0.98	−0.89
Complex II + III	61 (18–70)	0.82	0.16
Complex III	52 (42–123)	0.79	**−2.33**
Complex IV	60 (31–174)	0.82	−1.85
Citrate synthase	148 (50–122)	–	–

Legend: Activities are shown as nmol/min/mg protein. The control range (*n* = 30) is shown as (P5-P95). The ratios of the OXPHOS complexes over citrate synthase (Ratio/CS) after logarithmic transformation are calculated, with their respective Z-score. Z-scores lower than −1.96 are significantly different (*P* < 0.05) from the result in control samples and are indicative of a significantly decreased OXPHOS activity (shown in bold)

Single person Whole Exome Sequencing (WES) was performed and variants were filtered using a mitochondrial gene panel based on the Human MitoCarta2.0. dataset [16]. As the great majority of mitochondrial diseases follows a recessive mode of inheritance (after exclusion of mtDNA alterations), only variants that were homozygous or compound heterozygous were included. This filtering strategy resulted in two compound heterozygous variants in the *WARS2* gene (NM_015836). The first variant (c.797delC, p.Pro266ArgfsTer10) was a single nucleotide deletion causing a frameshift and premature stopcodon. The second variant (c.938 A > T, p.Lys313Met) was a missense variant. Both variants are rare in the healthy population, with respectively a prevalence of 3/121.378 and 18/121.412 individuals [17]. Both variants have recently been reported in compound heterozygous state in two subjects with WARS2 deficiency [10]. The second variant was also found in a heterozygous state with another pathogenic variant in two other reported subjects [9, 10]. Variants were confirmed by Sanger sequencing using variant-specific primers. Due to the close proximity of the two variants, *biallelic state* could be determined. A third primer pair was designed to confirm compound heterozygosity. This primer pair resulted in a PCR fragment containing both variants. As the position of the missense variant was not affected by the deletion, both variants were located on a different allele. Sanger sequencing of DNA from the mother, revealed that she is a carrier of the first variant (c.797delC, p.Pro266ArgfsTer10) and does not carry the missense variant. DNA from the father was not available for testing. Other genetic causes such as defects in *POLG*, *TWNK*, *mt- tRNA*^*Leu*^ and *mt- tRNA*^*Lys*^ were checked using the WES data and sequencing of the whole mt-DNA and chromosomal abnormalities were not detected.

### Western blot analysis

**Fig. 3:**
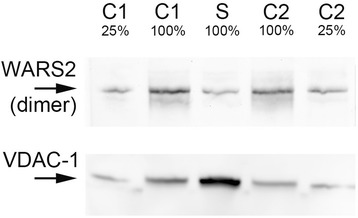
Immunoblotting of WARS2 and VDAC-1 in liver. Legend: Western blotting using antibodies against WARS2 and VDAC-1 in mitochondrial liver extracts from the proband and controls. C: controls and S: subject (proband)

Western blot analysis for WARS2 was performed in liver tissue from the proband and controls. Extracts were loaded in duplicate on the same gel and transferred to a nitrocellulose membrane. One part of the nitrocellulose membrane was incubated with WARS2 antibody and the second part with a cocktail of antibodies against subunits of the five OXPHOS complexes. After incubation with WARS2 antibody, a prominent band at approximately 90 kDa was seen in both controls. This band was clearly decreased in the proband when compared to controls (Fig. 3). WARS2 protein has a calculated molecular mass of around 40 kDa. The signal seen at 90 kDa was originating from the WARS2 homodimer [18]. The membrane was re-probed with a VDAC-1 antibody to check for equal loading (Fig. 3). The ratio of WARS2 signal over VDAC-1 signal shows an unequivocally decrease of the dimeric WARS2 protein in liver tissue from the proband.

Results from incubation with a cocktail of five antibodies (one against each of the five complexes) correlated with the results obtained by BN-PAGE. The intensity of the bands corresponding to complex I (NDFUB8) and complex IV (COX2) were decreased. The band corresponding to the complex II SDHB subunit was more intense in the proband. An increased signal of the complex V alpha subunit (54 kDa) was seen, which can be explained by the fact that following denaturing conditions, the complex V alpha signal is the combined signal of alpha subunits derived from the holocomplex and from the subcomplexes of complex V (data not shown).

## Discussion

Here, we report on a 6-year-old child with WARS2 deficiency. The proposita had presented at young age with developmental delay, motor weakness and intellectual disability. Her condition remained stable for several years until she presented for the first time with seizures at the age of six years and a treatment with sodium valproate was initiated. During the next month, her clinical condition deteriorated rapidly and ultimately she developed acute liver failure and severe encephalopathy. Sodium valproate was stopped but nevertheless she became progressively more unconscious and died half a year later.

WARS2 deficient subjects were only very recently reported in the literature. Musante et al. described the first two WARS2 deficient siblings in 2017. These two girls, 17 and 16 years old, had developmental delay (IQ 41–46), athetosis, speech impairment, motor weakness and unbalanced gait. [7]. Another WARS2 deficient male who was reported by Theissen et al. (2017) had developmental delay, seizures starting at the age of six months and severe language disability. At 24 years of age the neuromuscular phenotype included generalized amyotrophy, spastic quadriplegia, axial hypotonia, dysmetria, tremor and bilateral horizontal nystagmus. He died at age 24 years [9]. Six subjects with WARS2 deficiency were described by Wortmann et al. (2017). In three neonates overwhelming hyperlactacidemia was documented and fatal outcome occurred in early infancy. The fourth subject in this cohort presented at four months of age with hypotonia, severe cognitive and motor delay, cardiomyopathy and retinitis pigmentosa. He died at the age of three years. The two remaining subjects in this series presented with severe cognitive and motor delay, one at age 13 months and the other at 18 months. One of them had dystonia and the other ataxia, nystagmus and optic atrophy. Both were alive at, respectively, three years and ten years of age [10]. More recently, Burke et al. (2017) reported on a subject with a phenotype dominated by Parkinson-like signs. Development was normal until the age of one year, when a left leg tremor was first noticed. At 18 months of age, the tremor had become apparent also on the right side and in the upper extremities as well, along with intermittent dystonic posturing of all extremities. Treatment with Levodopa resulted in a stable period of 3–5 years with normal development regarding acquisition of motor, language and social milestones. Thereafter he began showing signs of more advanced Parkinson disease and neck dystonia [11].

Based on the clinical data of previously reported subjects, three different clinical phenotypes can be discerned, i.e. (a) a severe neonatal phenotype with overwhelming hyperlactacidemia and fatal outcome at very young age, (b) a more protracted course with early onset developmental delay, motor weakness, extra-pyramidal signs, with or without epilepsy, and (c) a phenotype characterized by normal early development and Parkinson-like symptoms starting around the age of one year. The phenotype of the subject reported here fits into the second phenotype (protracted course, early onset developmental delay, motor weakness, extra-pyramidal signs, epilepsy). She differs, however, from previously reported subjects as she developed acute onset liver failure. The hypothesis is that treatment with valproate has induced or triggered a severe hepatopathy. No other WARS2 deficient subjects with severe liver failure were reported previously. One subject, reported by Wortmann et al. was found to have hepatosplenomegaly which was attributed to a CMV infection. Later, liver function tests normalized in this subject [10]. An overview of the WARS2 deficient subjects is shown in Table 2.

**Table 2:** Clinical and molecular data of reported WARS2 deficient subjects

Subject Publication	Gender	Age at onset	Age at publication	Clinical findings	Molecular findings
F2-V:7 [7]	F	ND	17 years	Long philtrum, ataxia, athetosis, aggressive behaviour	Trp13Gly Ser109Alafs*15
F2-V:8 [7]	F	ND	16 years	Long philtrum, ataxia, athetosis, aggressive behaviour	Trp13Gly Ser109Alafs*15
[9]	M	Neonatal	24 years (deceased)	Severe infantile onset leukoencephalopathy, spastic quadriplegia, hypoglycemia (neonatal), epilepsy	Leu100del Lys313Met
F1, I1 [10]	F	Neonatal	23 days (deceased)	Fatal lactic acidosis	Lys31_Gln116del Val349Leu
F2, I2 [10]	M	Neonatal	3.5 years (deceased)	Lactic acidosis, epilepsy, hypoglycaemia (neonatal)	Pro266Argfs*10 Lys313Met
F2, I3 [10]	M	Neonatal	1.5 years (deceased)	Lactic acidosis, epilepsy, hypoglycaemia (neonatal)	Pro266Argfs*10 Lys313Met
F3, I4 [10]	F	4 months	3 years (deceased)	Epilepsy, muscular hypotonia, cardiomyopathy, retinitis pigmentosa	His77Gln Glu352Lys
F4, I5 [10]	M	13 months	3 years	Dystonia, suspected epilepsy	Val178Leu (homozygous)
F5, I6 [10]	F	18 months	10 years	Ataxia, nystagmus	Gly45Val Lys313Met
[11]	M	1 year	17 years	Parkinson-like symptoms, dystonia	Trp13Gly Ser228Trp
Proband	F	Before 6 months	6.5 years (deceased)	Epilepsy, ptosis, hypoglycaemia, valproate induced hepatotoxicity	Pro266Argfs*10 Lys313Met

Sodium valproate is known to have a potential toxic effect on mitochondrial functioning and is contraindicated whenever a mitochondrial defect is suspected in a subject. The pathogenesis of valproate toxicity is insufficiently understood. Valproate has been shown to inhibit the activity of the complexes I and IV. It inhibits oxygen consumption and adenosine triphosphate synthesis and sequestrates coenzyme A. It can disturb the structural organization of the inner mitochondrial membrane. Depletion of hepatic cytochrome *aa3* and inhibition of the mitochondrial beta-oxidation was documented [19, 20]. In most instances, the acute onset of liver failure occurs shortly after sodium valproate was initiated. Some of the affected subjects recover after discontinuation of the valproate treatment while in others the condition deteriorates further towards a fatal outcome. Most of the valproate induced hepatopathy subjects reported in the literature were carriers of pathogenic mutations in *POLG* [21–26]. Valproate toxicity was also documented in subjects with pathogenic mutations in *TWNK* [27]. It can aggravate epilepsy due to MELAS in subjects with the A3243G mutation in the mitochondrial DNA [28, 29] and can cause secondary carnitine deficiency in subjects with MERRF [30]. Fatal liver toxicity was also reported in a subject with CPEO (Chronic Progressive External Ophthalmoplegia) [31] and in several subjects with Alpers syndrome without genetic diagnosis [32, 33]. Spectrophotometric analysis in liver of most of the subjects showed the combined deficiency of complexes I, III and IV [25]. Sodium valproate can trigger a latent congenital liver disease that would otherwise have remained subclinical or would have manifested later in life.

In the proband, spectrophotometrical analysis and BN-PAGE in a liver biopsy specimen showed a deficient activity of complex III and low normal activities of the complexes I and IV. Complex V has two subunits that are encoded by the mtDNA. In case these subunits are not synthesized in the mitochondrial matrix, the rotor of the complex V (F1) can detach from the holo-complex, which can be detected as a smaller sub-complex in the BN-PAGE gel. As the ATP synthase activity of complex V is located in the alpha-subunit in the rotor, the presence of a sub-complex can be visualized in the BN-PAGE gel using the ATP-ase staining [34]. The presence of a subcomplex of complex V in our subject was therefore suggestive of an intramitochondrial protein synthesis defect. In earlier reported WARS2 deficient subjects, biochemical analysis in liver has not been performed. Immunohistochemical staining of OXPHOS enzymes in the liver of one subject reported by Wortmann et al. demonstrated severely reduced quantity of the complexes I and IV. Remarkably this subject did not have any clinical nor biochemical sign of liver failure. Immunohistochemical staining in his skeletal muscle was normal. OXPHOS activity measurements in skeletal muscle from four other subjects with WARS2 deficiency revealed mildly deficient enzymes in two of them [10, 11]. Activity testing in cultured skin fibroblasts was normal in all tested subjects in this series.

In the subject reported here, microscopic examination of liver showed a mosaic pattern after activity staining of cytochrome *c* oxidase. Hepatocytes with strong mitochondrial activity were found adjacent to cells that showed scarce or no reaction product (Fig. 1c-d). Mitochondrial mosaic staining for cytochrome *c* oxidase in liver has previously been reported in six subjects: (i) one with Alpers syndrome caused by pathogenic mutations in *POLG*, (ii) one with Pearson disease, (iii) in another subject with POLG deficiency [14], (iv) in an infant with encephalopathy and cholestatic giant cell hepatitis, (v) in a subject with fatal infantile hepatic failure due to mutations in *POLG* [26, 35], and (vi) in a subject with MEGDEL syndrome [36].

**Fig. 4:**
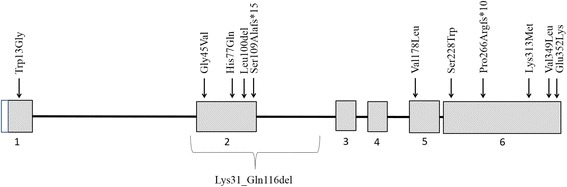
Localization of the 12 reported variants in *WARS2 (*NM_015836)

A defect in a nuclear encoded gene was suspected in the proposita as a combined defect of OXPHOS complexes involving the complexes I, III and IV was seen in liver, together with the presence of a subcomplex of complex V and the normal entire mtDNA sequencing. Single person WES was performed in order to pinpoint the underlying molecular defect. Two variants were found in *WARS2*. One was a frameshift mutation (c.797delC, p.Pro266ArgfsTer10), the other a missense mutation (c.938 A > T, p.Lys313Met). Both mutations are located in exon 6, the last exon of *WARS2* (NM_015836) (Fig. 4). Twelve different pathogenic mutations in *WARS2* have been reported so far. The identical compound heterozygous mutant genotype (p.Pro266ArgfsTer10/ p.Lys313Met) was found in the two subjects with severe neonatal expression reported by Wortmann et al. (Table 2). The third subject with neonatal phenotype in the series reported by the same group was compound heterozygous for p.Lys31_Gln116del and Val349Leu [10]. The p.Lys313Met missense mutation is the most frequently detected pathogenic mutation in *WARS2*, at least in the European population, as it was detected in five of the eleven reported subjects (including the one reported here). Of the twelve documented mutations, most of them were situated in exon six (5/12) or in exon two (5/12). Nine of the twelve pathogenic mutations were missense mutations and two of the twelve frameshift mutations (Fig. 4).

A wide variety of brain MRI abnormalities were found in the WARS2 deficient subjects. In the subject reported by Theissen et al. (2017), a delay of myelination, diffuse cerebral atrophy and moderate symmetric ventriculomegaly was described [9]. Also, four of the subjects reported by Wortmann et al. (2017) were found to have MRI abnormalities in the brain. In one, absent myelinisation of the white matter was seen, in another white matter edema and frontal atrophy, in the third hypoxemic-ischemic basal ganglia lesions and in the fourth cerebral and cerebellar atrophy [10]. In the subject reported by Burke et al. (2017) progressive generalized brain atrophy but no dysmyelination nor leukoencephalopathy or abnormalities of the basal ganglia were seen [11]. Cerebral MRI in the proband also showed atrophy most prominent in the frontal regions and, in addition, atrophy of the vermis cerebelli, brainstem and cerebellar peduncles.

## Conclusion

In conclusion, we broaden the clinical spectrum of WARS2 deficiency and report for the first time a severe hepatopathy associated with valproate treatment in a subject with WARS2 deficiency. Interestingly, the proband was initially considered as having a non-specific, non-progressive encephalopathy, and it was only when she developed hepatopathy after valproate treatment was started that the diagnosis of a mitochondrial defect was made. This report shows that one should be cautious when starting valproate treatment, even when a mitochondrial defect is not suspected and POLG deficiency is excluded. More observations are needed to confirm that WARS2 deficiency can be added to the list of mitochondrial defects associated with valproate-induced hepatopathy. Apparently, pathogenic mutations in the nuclear genes *POLG*, *TWNK* and *WARS2*, and in the mitochondrial genes *tRNA*^*Leu*^ and *tRNA*^*Lys*^, all affecting intramitochondrial transcription and/or translation, have been associated with valproate-induced acute liver failure.

## Abbreviations

BN-PAGE: Blue native polyacrylamide gel-electrophoresis
mt-aaRSs: Mitochondrial aminoacyl-tRNA synthetases
mtDNA: Mitochondrial DNA
OXPHOS: Oxidative phosphorylation
PAS: Periodic acid-Schiff
WES: Whole Exome Sequencing
